# Evaluation of Ceramics Adsorption Filter as a Pretreatment for Seawater Reverse-Osmosis Desalination

**DOI:** 10.3390/membranes12121209

**Published:** 2022-11-29

**Authors:** Jingwei Wang, Lee Nuang Sim, Jia Shin Ho, Keiko Nakano, Yusuke Kinoshita, Kenichiro Sekiguchi, Tzyy Haur Chong

**Affiliations:** 1 Singapore Membrane Technology Centre, Nanyang Environment and Water Research Institute, Nanyang Technological University, Singapore 637141, Singapore; 2 State Key Laboratory of Water Environment Simulation, School of Environment, Beijing Normal University, Beijing 100875, China; 3 Material Solution, Hitachi Metals Singapore Pte. Ltd., Singapore 629656, Singapore; 4 Kuwana Works, Hitachi Metals Ltd., Mie-ken 511-8511, Japan; 5 Metallurgical Research Laboratory, Hitachi Metals Ltd., Tochigi-ken 321-4367, Japan; 6 School of Civil and Environmental Engineering, Nanyang Technological University, Singapore 639798, Singapore

**Keywords:** seawater reverse osmosis, membrane fouling, desalination, autopsy analysis, adsorption

## Abstract

Seawater reverse osmosis (SWRO) is the most energy-efficient process for desalination to produce drinking water from seawater. However, its sustainability is still challenged by membrane fouling. Appropriate feed water quality is one of the crucial prerequisites for SWRO operation. In the current study, a ceramic adsorption filter (CAF), which was predominantly coated with an aluminum-based adsorbent (i.e., Alumina, Al_2_O_3_), was employed to enhance the pretreatment performance of SWRO. The fouling performance of SWRO pre-treated with a CAF was evaluated by feeding with real ultrafiltration (UF)-filtrated seawater collected from a seawater desalination R&D facility in Singapore. The flux decline profile showed that the presence of CAF after UF could mitigate around 10–30% of SWRO fouling. Based on the autopsy of the fouled SWRO membranes, it was observed that SWRO with CAF pre-treatment and daily regeneration could alleviate around 77.5% of Ca-induced inorganic fouling as well as 76% of lower biofouling. The present work highlights the potential of applying adsorption technology to enhance pre-treatment performance to extend the lifespan of SWRO membranes. Coupling the adsorbents on a ceramic filter should be a useful way to ease their implementation, i.e., inline adsorption and re-generation.

## 1. Introduction

Desalinated water is an important water source for overcoming water scarcity. It has been reported that more than 300 million people depend on desalinated water for their livelihood [1]. Seawater reverse osmosis (SWRO) has been acknowledged as the leading technology for desalination, due to its energy-efficient and cost-effective properties [1,2]. However, membrane fouling—caused by either the deposition of solutes or the growth of microorganisms—is still a serious issue that commonly occurs in pilot-scale SWRO plants. The presence of fouling not only induces extra costs in SWRO—i.e., expenditure on membrane chemical cleaning—but also deteriorates the produced water quality, which should be tackled carefully. Notably, to alleviate fouling, the feed water quality for SWRO is strictly controlled by pre-treatments. It should be worth noting that around 15–30% of the total cost of SWRO is associated with pretreatments prior to RO and the disposal of brine [1]. Advancing low-cost but high-efficiency pre-treatment technologies is thereby extremely valuable for SWRO to sustain drinking water production from desalination [3,4].

The successful operation of a SWRO plant strongly depends on the proper design and operation of the pre-treatment processes. Pre-treatment processes serve as the first barrier to remove contaminants in the seawater and to protect the downstream RO systems from severe fouling and scaling [5]. Generally, the pre-treatment processes for SWRO can be categorized into conventional and membrane-based treatments as well as combinations of these methods. The conventional treatment processes include coagulation-flocculation, dissolved air flotation (DAF), granular media filtration, sand filtration, and cartridge filtration; whereas membrane-based processes include microfiltration (MF) and ultrafiltration (UF) [4,6,7], as well as recent advancements in using nanofiltration [8]. Other emerging pre-treatment processes aiming to remove algal organic matter (AOM) or assimilable organic carbon (AOC) include gravity-driven membrane filtration [9,10,11,12], biofiltration [13], the combination of granular activated carbon filtration with UF [14], and ceramic membranes [15,16]. Even though MF/UF pre-treatment is commonly installed in most industrial-scale SWRO plants, serious fouling can still be found due to the poor retention properties of MF/UF for the solutes, i.e., dissolved organic carbon (DOC), and the ions that contributes to scaling [10,17,18]. Furthermore, these dissolved organics cannot only be deposited directly onto RO membrane surfaces to form organic fouling, but also serve as the conditioning layer or food source for microbial fouling. As such, SWRO usually suffers severe fouling—especially during the algal bloom season [14,17,19]—and therefore chemical cleaning is frequently employed. Therefore, SWRO development urgently needs advances in dissolved pollutant removal from raw seawater to strengthen its sustainability.

An effective approach for reducing the mass of solutes in SWRO feed is through adsorption. However, due to the massive salts in water, the selectivity of adsorbents is especially important—otherwise, the adsorption sites are readily occupied by NaCl. In this case, metal oxide adsorbents might be more suitable than carbon-based materials (i.e., carbon nanotubes), which have poor selective properties in salt environments [20]. Kim et al. (2017) proposed the incorporation of powdered iron oxide into the UF system for AOM removal. The study showed that iron oxide was able to achieve 50% removal efficiency of DOC and hence significantly reduced fouling in SWRO [20]. Aluminum oxide is another common adsorbent candidate for the removal of contaminants such as toxic metal ions, organic dyes, pesticides, and natural organic matter (NOM) in water [21,22]. However, the application of adsorbents still faces the issue of their use patterns. A feasible installation together with easy regeneration is plausible for industry use.

In this study, a ceramic adsorption filter (CAF) that was predominantly coated with an aluminum-based adsorbent (i.e., Alumina, Al_2_O_3_) was employed as a pre-treatment prior to SWRO, which was targeted at adsorbing organics in seawater. The durable lifespan of the SWRO was evaluated by feeding with real UF-filtrated seawater collected from a seawater desalination R&D facility in Singapore. The fouled SWRO membranes (i.e., with and without CAF treatment) were also autopsied to unveil the underlying fouling mitigation mechanisms. The achieved results shed light on the approach of applying adsorption technology to enhance the efficiency of SWRO pretreatment for the alleviation of membrane fouling.

## 2. Materials and Methods

### 2.1. Materials

**Figure 1:**
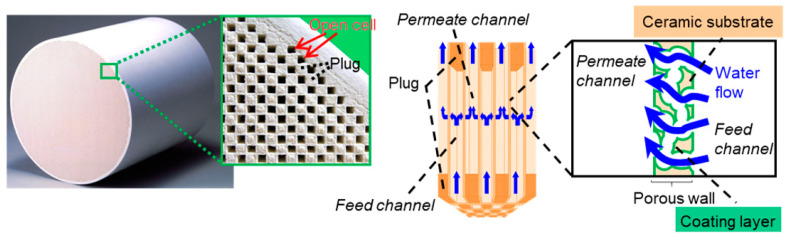
Photography of the CAF structure and diagram of the water flow path in CAF. Adapted from [23].

The ceramics adsorption filter (CAF) was provided by Hitachi Metals Ltd. (Japan); its details are exhibited in Figure 1. The surface of the alumina was positively charged at the pH of seawater (~pH 8); therefore, it had the tendency to adsorb negatively charged compounds in seawater—organic matter in particular. Compared to other ceramic membrane filters, this CAF is advantageous in terms of: (i) the high porosity of its walls (i.e., ~70%), (ii) its high chemical resistance, which allows periodical chemical cleaning, (iii) alumina particles with a particle diameter smaller than 1 µm were coated in the wall, which enabled adsorption on the large surface area provided from its microstructure in the coating layer, and (iv) its low hydraulic pressure loss—i.e., lower than 1 kPa—at the standard flow rate.

### 2.2. Experimental Setup

**Figure 2:**
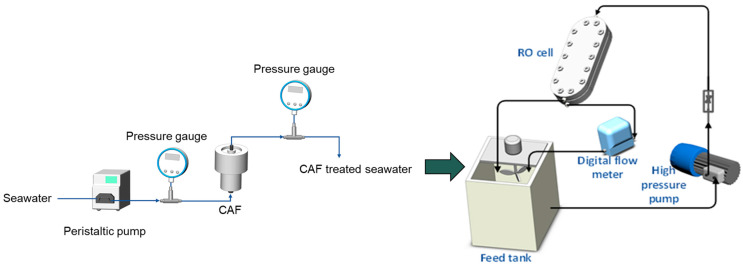
Schematic of the experimental setup with CAF pre-treatment.

Two lab RO systems were operated in parallel, one without (UF-RO) and another with a CAF (UF-CAF-RO) pre-treatment prior to RO, as shown in Figure 2. Specifically, a high-pressure pump (Hydra-Cell model D03) was used to pump the RO feedwater from a feed tank (around 15 L) to the crossflow RO cell at a flow rate of 0.5 L/min (corresponding to a crossflow velocity of 0.1 m/s). The flowrate was monitored using a rotameter with a range of 0.1–1 L/min (WF WATERFLO, China). The feed pressure of 45 bar (4.5 MPa) was regulated by a pressure regulator (Swagelok, Singapore) located after the RO filtration cell. The stainless-steel RO cell had an effective membrane area of 0.018 m^2^. The RO membrane used was a Nitto-Hydranautics SWC5-4040 fabricated with composite polyamide. Provided by the vendor, this RO membrane had high salt rejection (~99.8%) under 55 bar operation when treating simulated seawater (i.e., 32 g/L NaCl). Membrane flux (J) was calculated based on the permeate flow rate, as measured by a mass flowmeter (Brooks Instrument, model 5882). The retentate and permeate were fully recycled to the feed tank to maintain a constant volume. The conductivity of the feed and permeate were monitored with a conductivity transmitter (Thermo Fischer, model Alpha COND500). Values for the pressure, flowrate, and conductivity of the feed and permeate were recorded every 1 minute by CX Programmer software. The temperature of the RO feedwater in the feed tank was controlled at 28 °C by a chiller (PolyScience).

The UF-filtrated seawater collected from a seawater desalination R&D facility in Singapore was de-chlorinated with sodium bisulfite (i.e., a mixture of Na_2_S_2_O_5_ and NaHSO_3_) prior to use to prevent damage to the RO membranes. The de-chlorinated UF-filtrated seawater was used as RO feedwater in the UF-RO while it was further pre-treated with CAF in the UF-CAF-RO. The RO feedwater in both feed tanks was replenished on a daily basis during the experiment. 

For the system with the CAF pre-treatment, one unit of the CAF—with a total weight of around 24 g—was installed in plastic housing for usage. A constant feed flowrate of 70 mL/min was maintained using a peristaltic pump (Cole-Parmer, model 7522-20), equivalent to a space velocity of 120/h [23]. As shown in Figure 1, a CAF has an array of square flow channels (1.9 mm). The flow channels have porous walls that are coated with alumina particles (adsorbent). The flow channels are arranged to have an alternate pattern of ‘feed channel’ and ‘filtrate channel’. Each feed channel and filtrate channel has an open end and a non-porous ceramic plug on the opposite end. The feed water enters the bottom of the CAF via the feed channels and permeates through the porous wall, and then the filtrate exits the top of the CAF via the filtrate channels. A total volume of 10 L seawater was treated daily by the CAF. 

Two tests were carried out, known as the 1st and 2nd evaluations, which differed in their CAF backwash frequencies, i.e., 1st evaluation: the CAF was backwashed weekly, and 2nd evaluation: the CAF was backwashed daily. Specifically, the CAF was regenerated by backwashing after 7 days of treatment for the 1st evaluation, and regenerated daily after each treatment for the 2nd evaluation. It is worth noting that a reduced backwash frequency is important to (i) minimize the costs incurred by chemical cleaning and (ii) reduce the threat to permeate water quality posed by chemical additives. 

The backwash was conducted using 0.1 mol/L HCl and 0.1 mol/L NaOH solutions, respectively, at the same flow rate of 70 mL/min—whereby the cleaning solution was pumped in the opposite direction from the outlet for 2 min, then held for 10 min, followed by 2 min of flushing with DI water.

### 2.3. Water Quality Analysis

**Figure 3:**
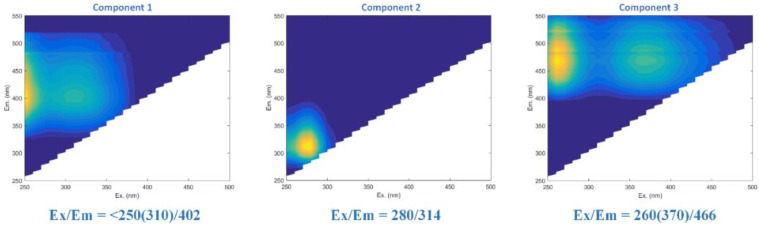
Components (i.e., C1, C2, and C3) identified using the EEM-PAPARAFAC method for the 2nd evaluation.

Samples (50 mL) were collected daily from various locations (i.e., before CAF, CAF-treated, RO feed tanks, and permeate) for measurements. The ion concentrations were measured using inductively coupled plasma optical emission spectroscopy (ICP–OES, PerkinElmer, model Optima8000). The dissolved organic concentration was measured in terms of total organic carbon (TOC) using a TOC analyzer (Shimadzu, model TOC-VCSH). The organic components were characterized by a Liquid Chromatography-Organic Carbon Detector (LC-OCD) Analyzer (DOC-Labor, Model 8)—Refer to reference [24]; the LC-OCD instrument can separate organic components into fractions, including humic substances, biopolymers, building blocks, low molecular weight organic acids, and neutrals based on their molecular weights. Further analyses of organic compounds were carried out using a fluorescence spectrophotometer (Agilent Technologies, Cary Eclipse). A statistical modelling method, namely PARAFAC, was coupled to further decompose the Excitation Emission Matrix (EEM) into individual components with unique fluorescence features using Matlab R2018b software with the DOMFluor toolbox, following the protocols described by Stedmon and Bro [25]. Specifically, the fluorescence spectra corresponding to the blank (Milli-Q water) was subtracted from each measured EEM, and the fluorescence intensities were further normalized by the Raman peak of Milli-Q water at an excitation wavelength of 350 nm and were calibrated against the quinine sulfate (QS) dilution series, producing EEMs in units of quinine sulfate equivalent (µg/L QSE). Three EEM components (C1, C2, and C3) were successfully extracted via the EEM-PARAFAC method, as shown in Figure 3. According to references [26,27,28], C1, C2, and C3 were identified as marine humic-like substances, amino acids or proteins, and terrestrial humic-like substances, respectively. 

### 2.4. Membrane Autopsy Analysis Methodology

At the end of the experiments, the fouled SWRO membrane was cut into three sections along the flow direction (i.e., lead, middle, and tail) for the analysis, and the result of their average was used to represent the whole RO membrane. Specifically, two pieces of fouled membrane with an area of 3 × 3 cm were cut from the lead, middle, and tail section, respectively, and were then soaked in 35 mL of sterilized 0.85% NaCl solution and sonicated for 5 min following 10 s of vortexing (Velp Scientifica, model RX3) for the detachment of foulants from the membrane surfaces. The extracted fouling solutions were used for the organic characterization and microbial analysis.

#### 2.4.1. Extracellular Polysaccharide Substances (EPS)

EPS were measured by the sum of the contents of polysaccharide and protein. The polysaccharide content was measured using the colorimetric method. Specifically, 2 mL of sample solution was mixed with 1 mL of 0.5% (*v*/*v*) phenol solution and 5 mL of concentrated H_2_SO_4_. The solutions were then left to cool down to room temperature for 15 min. Subsequently, the UV absorbance at 490 nm (A_490_) was measured using a UV spectrometer. The standard calibration curve was measured using glucose (Merck, Singapore). The quantification of the protein content in the EPS was conducted using a Micro Bicinchoninic acid (BCA) Protein Assay Kit (Pierce, #23235). Specifically, 2 mL of the working solution was added to 1 mL of sample solution. The mixtures were incubated in the dark at room temperature for 2 h. Then, the UV absorbance was measured at 562 nm (A_562_). The standard calibration curve was constructed using Bovine serum albumin (BSA, Pierce). 

#### 2.4.2. Dissolved Organics

The dissolved organics were obtained by filtering the mixtures with a 0.45 µm PES syringe filter. The detailed DOC characterization includes the TOC concentration, EEM, and LC-OCD.

#### 2.4.3. Cell Viability

The extracted foulant mixture was first passed through a 40 µm filter. The cell viability was quantified using a flow cytometer (BD, USA). Specifically, 1 mL of sample was stained with 1 µL of SYTO9 and PI each (Molecular Probes, USA). The sample was transferred to a flat-bottom well-plate for flow cytometry analysis (with an unstained sample as a control). Proprietary software (CSampler, BD, USA) was used to process the results. Counts in a defined region of a density plot were converted to live and dead cell counts.

#### 2.4.4. Field Emission Scanning Electron Microscopy (FESEM)

The fouled RO membrane was oven-dried at 50 °C for 24 h and sputter coated with platinum using a JFC-1600 auto fine coater before the SEM analysis. Field emission scanning electron microscopy (FESEM, JSM-7600F, JEOL) was utilized for the characterization of fouled membranes.

#### 2.4.5. Other Measurements

Surface functional groups were characterized by Fourier-transform Infrared spectroscopy (FTIR, Shimadzu, IRPrestige-21). Ion levels fouled on the RO membrane surfaces were quantified by Inductively Coupled Plasma–Optical Emission Spectroscopy (ICP–OES) (Perkin Elmer Optima 8000).

### 2.5. CAF Autopsy Analysis Methodology

The CAF autopsy was conducted by breaking the CAF into small specimens at the end of the filtration test. Three specimens along the flow direction (i.e., lead, middle, and tail) were selected for the analysis, and the result of their average was used to represent the whole CAF. Specifically, the foulants were extracted by soaking the CAF pieces in 35 mL sterilized 0.85% NaCl solution before sonication for 5 min following 10 s vortexing (Velp Scientifica). The autopsy analysis included the measurement of bacteria cell count per g CAF and adsorbed organic and inorganic amounts per g CAF.

## 3. Results and Discussion

### 3.1. RO Membrane Performance

**Figure 4:**
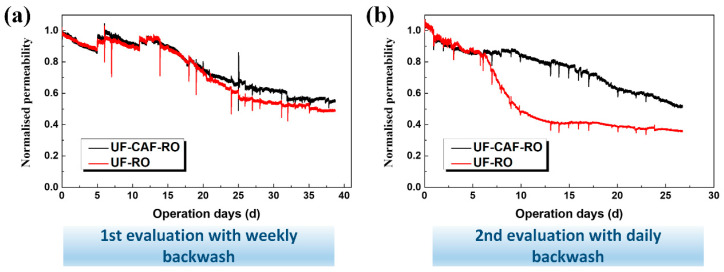
Evolution of the normalized permeability of RO membranes with and without CAF treatment.

Figure 4 depicts the evolution of the normalized permeability of RO with and without CAF treatment. To ease the comparison, normalized permeability rather than the raw data was presented, which was calculated by the ratio of permeability at time t to the one at the initial time of 0 s, i.e., permeability (t)/permeability (t = 0). The organic and salt rejections of RO were >98% and did not show a difference between UF-CAF-RO and UF-RO systems. Two observations are worth noting from Figure 4: Firstly, the normalized permeability showed a decreasing trend with operating time, indicating the occurrence of membrane fouling. The decrease in permeate flux might be ascribed to the growth of a biofilm or precipitations on the RO membrane surface [29], and will be further explored by the autopsy analysis. In particular, there appeared to be a sharp drop in flux for the UF-RO in the 2nd evaluation (Figure 4b). As the quality of seawater collected from the R&D facility seasonally changed, this sharp drop was presumably due to the sudden change in water quality to poor, giving excess burden to RO desalination. Secondly, the UF-CAF-RO exhibited higher permeability (i.e., less membrane fouling) compared to the UF-RO. The normalized permeability difference was enlarged from 10% to 30% when a higher frequency of CAF backwash was adopted, i.e., from weekly to daily—possibly due to the better removal of organics by the fresh than by the saturated CAF. The results in Figure 4 imply that CAF pretreatment prior to RO was able to alleviate RO membrane fouling, benefiting from the adsorption effect. The underlying fouling mitigation mechanism via alumina adsorption will be further explored in the following water quality and membrane autopsy analysis. 

### 3.2. Water Quality of the RO Feed

Table 1 summarizes the RO feed water quality of the UF-CAF-RO and UF-RO. As shown from Table 1, the average RO feed water quality with and without CAF treatment was quite similar—presumably due to the short contact time with the CAF in the single-pass configuration. However, a big difference was found for total bacteria levels. It was observed that the UF-CAF-RO had lower (~67.2%) numbers of bacteria cells compared to the UF-RO. The growth of the bacteria was inhibited by CAF pretreatment prior to RO. This presumably was due to the beneficial effect of CAF being able to remove some of the bacteria cells and assimilable organics from the seawater, thus alleviating the occurrence of biofouling on the RO membrane surface. The details of the CAF autopsy analysis in Section 3.4 have affirmed that CAF was able to remove the dead cells from seawater.

### 3.3. Membrane Autopsy Results

#### 3.3.1. Characterization of Dissolved Organic Matter (DOM) on Fouled RO Membranes

Table 2 gives a comparison of dissolved organic components on the fouled RO membranes with and without CAF treatment. The UF-CAF-RO membrane had a slightly higher level of organics than the UF-RO membrane, which was counter-intuitive as the flux of the UF-CAF-RO was higher (i.e., less fouling). These phenomena may be accounted for by two explanations: Firstly, the flux decline for the UF-RO system was faster due to the more severe membrane fouling and reaching the flux plateau stage earlier than the UF-CAF-RO system. The deposition of foulants such as macromolecular solutes were allayed at the final stages as the drag force weakened at low flux levels. Secondly, the severe flux decline of the UF-RO system was presumably caused by inorganic fouling and biofouling, which would be manifested by inorganics tests and microbial analyses.

The LC-OCD results showed that the dominant organic foulant was LMW organics due to the weak adsorption capacity of the CAF for low molecular weight organics. The component 2 (C2) protein-like substance was dominant on the fouled RO membranes rather than the humic acids (i.e., C1 and C3). The protein-like substances (C2) were possibly present in low molecular weight forms and could be generated by the bacteria’s activities or may have originated from the seawater. Therefore, a closer inspection of the bacterial growth on the membrane surfaces was important.

#### 3.3.2. Microbial Analysis of the Foulants

**Figure 5:**
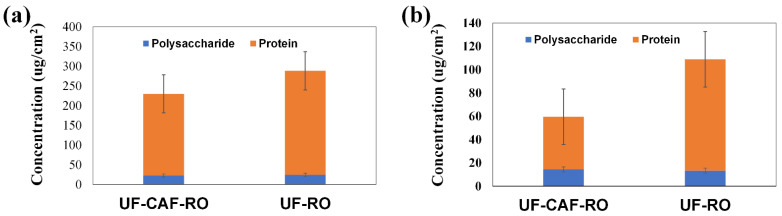
EPS content of the RO membranes with and without CAF pre-treatment in the (**a**) 1st evaluation and (**b**) 2nd evaluation.

The EPS content of the fouled RO membranes is shown in Figure 5. The EPS was mainly composed of protein rather than polysaccharide. The amount of EPS on the UF-CAF-RO fouled RO membrane was 20% and 45% lower than that on the UF-RO fouled RO membrane for the weekly and daily CAF backwash test, respectively. 

**Figure 6:**
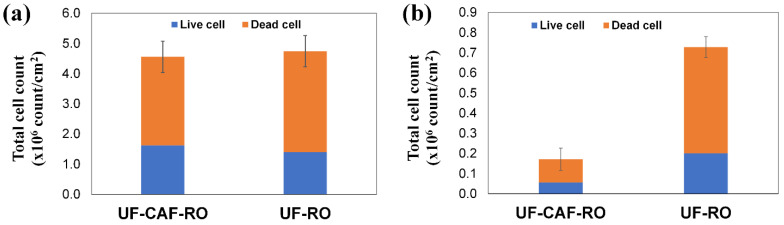
Live/dead cell count on the fouled RO membranes with and without CAF pre-filtration in the (**a**) 1st evaluation and (**b**) 2nd evaluation.

Figure 6 indicated that the cell count of the CAF-UF-RO was similar to that of the UF-RO fouled RO membrane in the 1st evaluation, but was 76% lower in the 2nd evaluation. This was possibly benefited by the presence of the CAF removing dead bacteria in the single-pass filtration mode, as demonstrated above in Section 3.2. The presence of the CAF seemed to suppress bio-accumulation on the RO membrane.

A detailed analysis of the organics from the autopsy suggested that organics might not be the main contributor to the flux decline in the RO performance, but rather the growth of the bacteria on the membrane surface might have hindered the filtration performance.

#### 3.3.3. Inorganic Fouling and FTIR Results

As seen from Table 3, the calcium concentration was significantly lower in the UF-CAF-RO compared to the UF-RO (~77.5% lower). These results suggest that the CAF could reduce Ca-induced fouling in SWRO. The amount of magnesium fouling on the RO membranes was quite similar. The presence of the CAF exhibited a greater influence for Ca rather than Mg-induced fouling. It should be worth noting that the inorganic fouling on the RO membranes was dominated by the precipitates, i.e., CaCO_3_, CaSO_4_, MgCO_3_, etc. The CAF has a positive surface zeta potential value [23], which would help to remove some of the anion ions such as CO_3_^2-^, and SO_4_^2-^—alleviating the inorganic precipitates. Considering that the solubility of Mg-formed precipitates (e.g., MgCO_3_) is higher than that of Ca, the effects of the CAF on inorganic fouling mitigation were more obvious for Ca-induced foulants. The Al and Si amounts on the UF-CAF-RO membrane were slightly higher than on the UF-RO, presumably due to the fragments released from CAF surfaces. However, the deposition amounts of Al and Si were very low, which should not influence the RO performance.

**Figure 7:**
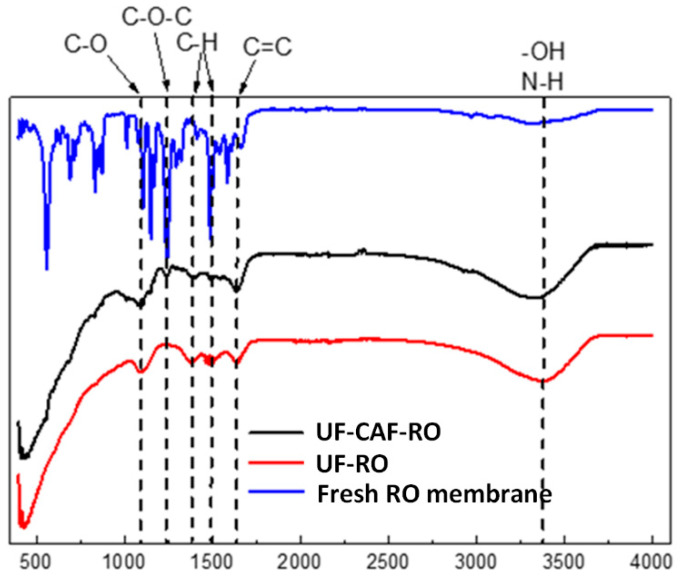
ATR-FTIR figures for the fouled RO membrane in the 2nd evaluation.

Figure 7 shows the FTIR spectrum of the fouled RO membrane. The main peaks were in the vicinity of 3387 cm^−1^ (O–H stretching and N–H stretching), 1103 (C–O stretching of polysaccharides), 1379 (phenolics), and 1645 (C=O stretching of amide I and C–N amide I attributed to the presence of proteins). The results further indicated the presence of organic fouling on the RO membranes—especially the protein and polysaccharide-induced fouling.

#### 3.3.4. Morphology Characterization

**Figure 8:**
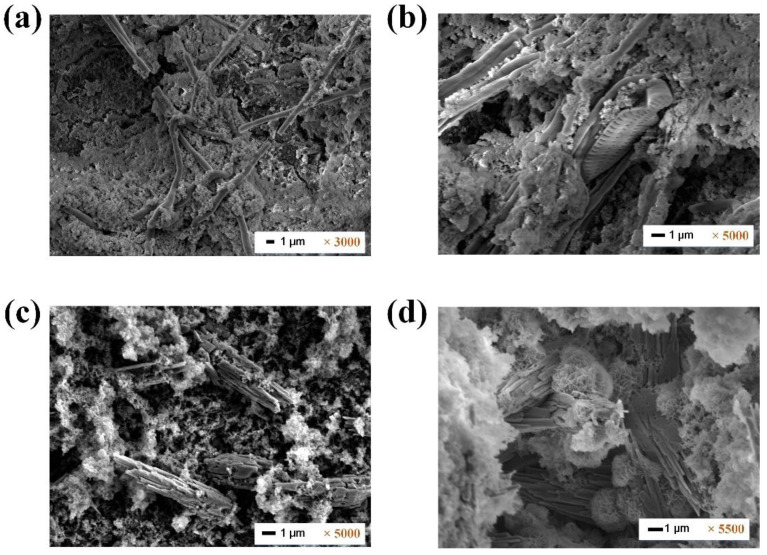
FESEM images of the RO fouled membrane surfaces (**a**,**b**) with CAF pretreatment and (**c**,**d**) without CAF pre-treatment from the 2nd evaluation (i.e., with daily CAF backwash).

**Figure 9:**
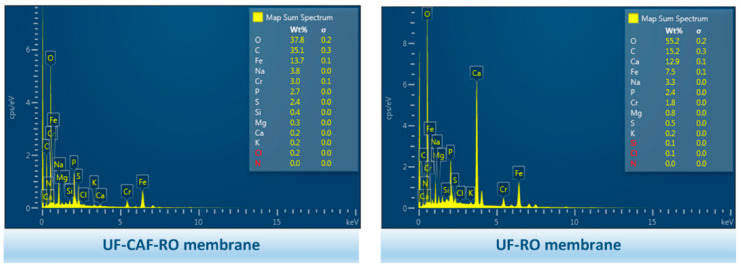
EDX analysis results of the fouled membrane surfaces from the 2nd evaluation (i.e., daily CAF backwash).

As seen from the SEM images in Figure 8, the RO fouling layer was composed of organic, inorganic, and biofouling. However, there appeared to be significant differences between the UF-CAF-RO and UF-RO fouled membrane surfaces, which suggested different fouling mechanisms. Specifically, the fouling on the RO membrane surfaces associated with CAF pretreatment (Figure 8a,b) formed thick cake layers embedded with several thin strips, while the RO membrane surfaces associated with the UF-RO formed inhomogeneous fouling layers with several significant crystallizations. It has been demonstrated that spider web-type foulants (Figure 8a,b) are attributed to the presence of biopolymers [29,30], i.e., the accumulation of EPS, which was further affirmed by the EDX analysis in Figure 9. Moreover, some microorganisms were found in the fouling layers, as shown in Figure 8b,d, implying the growth of bacteria that contribute to biofouling. The SEM images in Figure 8 affirmed the aforementioned fouling phenomenon and that serious inorganic fouling and biofouling were found on RO membranes, which primarily influenced the RO membrane flux.

From the SEM and EDX results, the inorganic fouling was dominated by the Ca and Mg; however, the accumulation of Ca and Mg was negligible on the UF-CAF-RO membrane surface. Considering the significantly high O content in the EDX element maps, the calcium was possibly due to the formation of CaCO_3_. In addition, a certain amount of Fe, which was ascribed to rust, was identified in Figure 9. Due to the lower content of inorganic fouling in the UF-CAF-RO, the associated Fe weight percentage was higher on the UF-CAF-RO membrane surface compared with the UF-RO membrane. The content of other elements, i.e., Na, P, S, Si, K, Cl, and N, etc., on the fouled RO membranes was similarly low (i.e., <4%) for both the UF-RO and UF-CAF-RO systems—indicating the negligible fouling of components formed by these elements.

### 3.4. CAF Autopsy Results

Table 4 shows the level of organics, inorganics, and live/dead cells accumulated on the CAF surface after the 2nd evaluation operation. In general, (i) a higher amount of protein-like organics (0.026 µg QSE/g CAF) were found compared to marine humic materials and microbial-derived compounds and terrestrial humic-like substances; (ii) a higher accumulation of dead cells than live cells was found, which contributed to the protein-like organics on the CAF surfaces. These dead cells resulted from the chlorination of seawater at the intake. In the meantime, the CAF had significant adsorption effects on the removal of inorganic matter—i.e., Na, Mg, and Ca—and had a relatively lower adsorption capacity for DOC, which could also support the explanation of the membrane fouling mechanism identified by the membrane autopsy, i.e., inorganic fouling and biofilms hampering the membrane flux. 

## 4. Conclusions

The presence of CAF pre-treatment prior to SWRO showed positive effects on the downstream RO process. It is significant that the UF-CAF-RO always achieved higher permeability (i.e., less membrane fouling) compared to the UF-RO; this difference was enlarged from 10% to 30% with more frequent CAF backwashes, i.e., from weekly to daily. From the RO membrane autopsy analysis, the UF-CAF-RO was found to have less inorganic scaling, a lower EPS content, and less bacteria accumulation compared to the UF-RO. The inorganic fouling was dominated by Ca in the form of CaCO_3_. 

Although the water quality between the UF-CAF-RO and UF-RO was similar, the pollutants fouled on the RO membrane surfaces were significantly different. This might be attributed to (i) the mass removed by CAF filtration being less but sufficient to differentiate the fouling performance of SWRO; (ii) the CAF being able to adsorb the organics that were attractive for microorganisms—thus alleviating the EPS amount by inhibiting the bacterial growth on the RO membranes, which could be affirmed by the lower cell count results; (iii) the CAF being able to alleviate the formation of precipitates, i.e., CaCO_3_, as the surface zeta potential was positive at the seawater pH range.

The current work proposed an effective alternative for SWRO pretreatment using an adsorption method, whereby the CAF was selected. When adsorption saturation was achieved, the CAF could be regenerated by inline chemical cleaning. To facilitate its implementation, further advances should include an understanding of CAF adsorption mechanisms, the optimization of CAF usage, and the proper design of CAF streams for easier regeneration. 

## Figures and Tables

**Table 1:** Water quality of RO feed water for UF-CAF-RO and UF-RO.

	1st Evaluation		2nd Evaluation	
**Parameters**	**UF-CAF-RO**	**UF-RO**	**UF-CAF-RO**	**UF-RO**
TOC (ppb)	1321 ± 182	1362 ± 309	1421 ± 139	1405 ± 128
BP (ppb)	N.M.	N.M.	78 ± 4	48 ± 24
HA (ppb)	N.M.	N.M.	475 ± 17	420 ± 34
BB (ppb)	N.M.	N.M.	188 ± 7	175 ± 9
LMW (ppb)	N.M.	N.M.	388 ± 106	294 ± 119
Na (ppm)	8527 ± 249	8423 ± 226	9757 ± 1523	10,129 ± 1638
Mg (ppm)	1281 ± 36	1269 ± 18	2599 ± 182	2451 ± 368
Ca (ppm)	330 ± 8	327 ± 4	556 ± 35	526 ± 73
B (ppm)	N.M.	N.M.	3.6 ± 0.16	3.7 ± 0.06
C1-Marine HA (µgQSE/L)	N.M.	N.M.	7.44 ± 1.47	6.61 ± 1.00
C2-Proteins (µgQSE/L)	N.M.	N.M.	4.75 ± 0.52	4.14 ± 0.83
C3-Terrestrial HA (µgQSE/L)	N.M.	N.M.	3.40 ± 0.77	3.33 ± 0.39
Total bacteria amount (count/µL)	N.M.	N.M.	4.2 ± 3.7	12.8 ± 8.3

Note: N.M. means not measured. BP: Biopolymer; HA: Humic acid; BB: building block; LMW: Low molecular weight substances.

**Table 2:** Characterization of dissolved organic components on fouled RO membranes.

	1st Evaluation			2nd Evaluation		
Parameters	UF-CAF-RO	UF-RO	Compared with UF-RO	UF-CAF-RO	UF-RO	Compared with UF-RO
DOC (µg/cm^2^)	2.463	2.247	9.6% higher	2.450	1.878	30.5% higher
BP (µg/cm^2^)	N.M.	N.M.	N.M.	0.295	0.200	47.5% higher
HA (µg/cm^2^)	N.M.	N.M.	N.M.	0.098	0.084	16.7% higher
BB (µg/cm^2^)	N.M.	N.M.	N.M.	0.103	0.051	102.0% higher
LMW (µg/cm^2^)	N.M.	N.M.	N.M.	1.055	0.461	128.9% higher
C1-Marine HA (µgQSE/cm^2^)	N.M.	N.M.	N.M.	0.007	0.006	16.7% higher
C2-Proteins (µgQSE/cm^2^)	N.M.	N.M.	N.M.	0.050	0.035	42.9% higher
C3-Terrestrial HA (µgQSE/cm^2^)	N.M.	N.M.	N.M.	0.003	0.002	50.0% higher

Note: N.M. means not measured.

**Table 3:** Comparative analysis of the inorganic ions fouled on the RO membranes in the 2nd evaluation.

Parameters	UF-CAF-RO	UF-RO
Na (µg/cm^2^)	1326.11	1321.11
Mg (µg/cm^2^)	34.16	34.01
Ca (µg/cm^2^)	9.19	40.84
B (µg/cm^2^)	0.90	0.87
Al (µg/cm^2^)	0.06	0.00
Si (µg/cm^2^)	0.25	0.11

**Table 4:** Autopsy analysis results for the CAF after the 2nd evaluation operation.

Parameters	CAF Adsorption Amount
DOC (µg/g CAF)	4.7
Na (µg/g CAF)	3411
Mg (µg/g CAF)	597
Ca (µg/g CAF)	143
B (µg/g CAF)	3.9
C1-Marine HA (µg QSE/g CAF)	0.0069
C2- Proteins (µg QSE/g CAF)	0.026
C3-Terrestrial (µg QSE/g CAF)	0.0020
Live cell (Count/g CAF)	0.32 × 10^6^
Dead cell (Count/g CAF)	3.16 × 10^6^

## Data Availability

Not applicable.
